# Supplementary material for: Spanish Cross-Cultural Adaptation and Validation of Neck Bournemouth Questionnaire (NBQ) for Neck Pain Patients
Source: Healthcare (Basel). 2023 Jul 3;11(13):1926. doi: 10.3390/healthcare11131926 (PMC10341303; doi:10.3390/healthcare11131926)
Supplement: Supplementary file 1 [file healthcare-11-01926-s001.zip › healthcare-2406218-Supplementary File S1.pdf]

NECK BOURNEMOUTH QUESTIONNAIRE PARA DOLOR CERVICAL

Nombre y apellido:

Fecha:

Las siguientes preguntas han sido diseñadas para conocer su dolor de cuello y cómo le está afectando. Por favor, responda a todas las preguntas marcando con un círculo un número en cada escala numérica que mejor describa cómo se siente:

1. Durante la última semana, ¿cómo calificarías de media tu dolor de cuello?

Sin dolor — Peor dolor posible

0 1 2 3 4 5 6 7 8 9 10

2. Durante la última semana, ¿en qué medida te ha molestado el dolor de cuello en tus actividades cotidianas (tareas domésticas, lavarse, vestirse, levantar peso, leer, conducir)?

Sin molestias — Imposibilidad de realizar actividades

0 1 2 3 4 5 6 7 8 9 10

3. Durante la última semana, ¿en qué medida ha interferido el dolor de cuello en tu capacidad para participar en actividades recreativas, sociales y familiares?

Sin molestias — Imposibilidad de realizar actividades

0 1 2 3 4 5 6 7 8 9 10

4. Durante la última semana, ¿hasta qué punto te has sentido ansioso/a (tenso/a, irritable, con dificultad para concentrarte/relajarte)?

Nada ansioso — Extremadamente ansioso

0 1 2 3 4 5 6 7 8 9 10

5. Durante la última semana, ¿cómo de deprimido (abatido, triste, desanimado, pesimista, infeliz) te has sentido?

Nada deprimido — Extremadamente deprimido

0 1 2 3 4 5 6 7 8 9 10

6. Durante la última semana, ¿has sentido que tu trabajo (tanto dentro como fuera de casa) ha afectado (o afectaría) a tu dolor de cuello?

No lo ha empeorado	Lo ha empeorado mucho

0	1	2	3	4	5	6	7	8	9	10

7. Durante la última semana, ¿hasta qué punto has podido controlar (reducir/ayudar) tu dolor de cuello por ti mismo?

Controlado completamente	Sin control alguno

0	1	2	3	4	5	6	7	8	9	10
